# ([2.2.2]Cryptand)potassium (4-methyl­benzene­thiol­ato)[5,10,15,20-tetra­kis­(4-chloro­phen­yl)porphyr­inato]manganate(II) tetra­hydro­furan disolvate

**DOI:** 10.1107/S2414314622002413

**Published:** 2022-03-08

**Authors:** Yiwen Yuan, Jianfeng Li

**Affiliations:** a College of Materials Science and Opto-electronic Technology, CAS Center for Excellence in Topological Quantum Computation & Center of, Materials Science and Optoelectronics Engineering, University of Chinese Academy of Sciences, Yanqi Lake, Huairou District, Beijing 101408, People’s Republic of China; Goethe-Universität Frankfurt, Germany

**Keywords:** crystal structure, Mn^II^ porphyrin, 4-methyl­benzene­thiol­ate

## Abstract

Single crystals of the title compound, [K(C_18_H_36_N_2_O_6_)][Mn(C_44_H_24_Cl_4_N_4_)(C_7_H_7_S)]·2C_4_H_8_O, were obtained by the solvent evaporation method. The Mn^II^ cation is coordinated by four pyrrole N atoms (N_p_) of the porphyrin ring and one S atom of the apical 4-methyl­benzene­thiol­ate ligand with the average Mn—N_p_ and the apical Mn—S bond lengths being 2.160 (9) and 2.4642 (8) Å, respectively. Two tetra­hydro­furan solvent mol­ecules and a potassium cation chelated inside a [2.2.2]cryptand (4,7,13,16,21,24-hexa­oxa-1,10-di­aza­bicyclo[8.8.8]hexa­cosa­ne) are also present.

## Abstract 2

The Mn^II^ centre is coordinated by four pyrrole N atoms of the porphyrin ring and one S atom of the axial 4-methyl­benzene­thiol­ate ligand. Two tetra­hydro­furan mol­ecules and a potassium cation chelated inside a [2.2.2]cryptand (4,7,13,16,21,24-hexa­oxa-1,10-di­aza­bicyclo­[8.8.8]hexa­cosa­ne) are also present.

## Graphical abstract

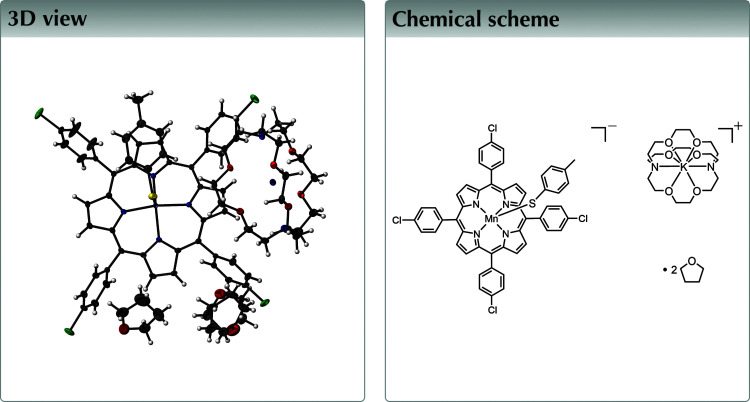

## Structure description

Mn^II^ porphyrin analogues are often used as substitutes and supplements for Fe^III^ porphyrin model compounds when simulating the relevant reactions of heme-active sites, so as to provide a more comprehensive understanding of structure–function relationships in hemoproteins (Gibson *et al.*, 1974 ▸; Hoffman *et al.*, 1975 ▸). In 1977, Scheidt and co-workers published the first crystal structure comprising a five-coordinate manganese porphyrin complex, [Mn(TPP)(1-MeIm)] (Kirner *et al.*, 1977 ▸). The high-spin (*S* = 5/2) [Mn(TPP)(1-MeIm)] has a large metal out-of-plane distance (Δ_24_ and Δ_4_ ≥ 0.51), and the authors suggested that when the *d*_x2–*y*2_ orbital is populated, the metal atom is too far out of the porphyrin plane to permit effective inter­action with a sixth ligand. Subsequently, some other crystal structures of five-coordinate manganese porphyrins were reported, *e.g.* [K(222)][Mn(TPP)(CN)] (TPP = *meso*-tetra­phenyl­porphyrin, CN = cyano; He *et al.*, 2016 ▸), and [K(222)][Mn(TPP)(4-MeIm^−^)] (4-MeIm^−^ = 4-methyl­imidazole anion; Zhao *et al.*, 2021 ▸). In this work, we report the determination of a new manganese(II) porphyrin crystal structure – [K(222)][Mn(T*p*ClPP)(*p*-CH_3_PhS^−^)].

**Figure 1:**
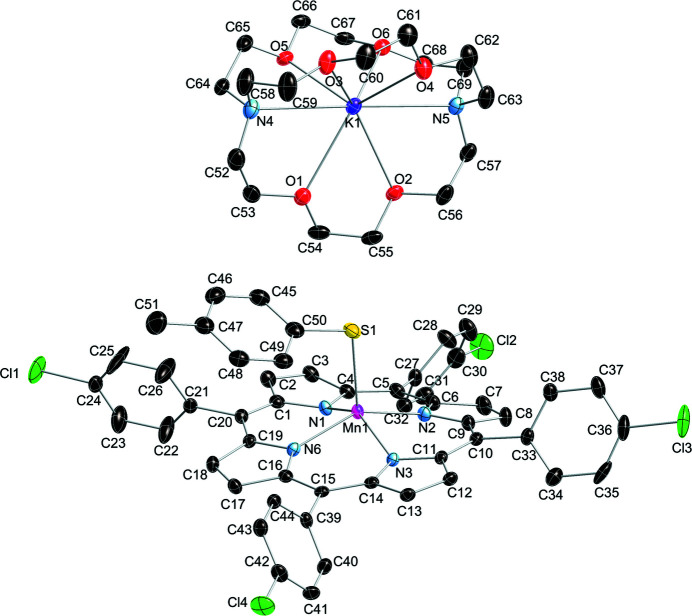
*ORTEP* diagram of the mol­ecular entities in the title compound with displacement ellipsoids drawn at the 50% probability level. The two tetra­hydro­furan solvent mol­ecules and H atoms have been omitted for clarity.

The mol­ecular entities of the title compound are shown in Fig. 1 ▸. In the title compound, the counter-ion to the negatively charged five-coordinate Mn^II^ porphyrinate, *i.e.* the [Mn^II^(T*p*ClPP)(*p*-CH_3_PhS^−^)]^−^ anion, is a [K(222)]^+^ cation in which the potassium ion is chelated inside a [2.2.2]cryptand mol­ecule. Six O and two N atoms of the [2.2.2]cryptand bind to the potassium cation, and the average K—O and K—N distances are 2.85 (4) and 2.989 (11) Å, respectively. There are also two tetra­hydro­furan (THF) solvent mol­ecules in the asymmetric unit, one of which is disordered.

It is inter­esting to note that Mn^II^ has a larger ionic radius than Fe^III^ (0.750 *versus* 0.645 Å; Shannon, 1976 ▸), although both ions are isoelectronic with *d* ^5^ electronic configurations. Hence, the title compound shows a larger metal displacement from the 24-atom mean plane [0.72 Å *versus* 0.45–0.51 Å), longer *M*—N_p_ (N_p_ is a porphyrin N atom) bond lengths [2.160 (9) Å *versus* 2.057 (5)–2.06 (12) Å], and a longer Mn—S apical distance [2.4642 (8) Å *versus* 2.298 (5)–2.311 (3) Å] (Byrn & Strouse, 1991 ▸; Miller & Strouse, 1984*a* ▸,*b* ▸).

## Synthesis and crystallization

**General information**

Unless otherwise noted, all experimental operations in this work were carried out under an argon (Ar) atmosphere using standard Schlenk techniques, and all solvents used were treated under anhydrous and anaerobic conditions. Benzene and tetra­hydro­furan (Sinopharm Chemical Reagent) were distilled over sodium/benzo­phenone. Hexane (Beijing Chemical Works) was distilled from sodium/potassium alloy under argon. Pyrrole, *p*-chloro­benzaldehyde, 2,6-dimeth­yl–pyridine, and 4-methyl­benzene­thiol were vacuum distilled under Ar and stored in a refrigerator for further use. *p*-CH_3_PhSK was prepared according to a published procedure (Hu *et al.*, 2010 ▸) except that the 2-methyl­imidazole was replaced with 4-methyl­benzene­thiol (Aladdin chemicals). H_2_T*p*ClPP, [Mn(T*p*ClPP)Cl], and [Mn(T*p*ClPP)OH] were prepared according to a literature method (Adler *et al.*, 1967 ▸, 1970 ▸; Fleischer & Srivastava, 1969 ▸). [Mn^II^(T*p*ClPP)] was prepared by reduction of [Mn^III^(T*p*ClPP)OH] (10 mg, 0.0012 mmol) with ethane­thiol (1.5 ml) in benzene (5 ml) (Stolzenberg *et al.*, 1981 ▸).

**Synthesis of ([2.2.2]cryptand)potassium (4-methyl­benzene­thiol­ato)[5,10,15,20-tetra­kis­(4-chloro­phen­yl)porphyrinato]mang­anese(II) tetra­hydro­furan disolvate**

The purple [Mn^II^(T*p*ClPP)] (10 mg, 0.0012 mmol) powder was dried under vacuum and then dissolved in 5 ml of tetra­hydro­furan. 6.0 mg (0.050 mmol) of *p*-CH_3_PhSK and 17.0 mg (0.045 mmol) of Kryptofix 222 were added to the solution. The mixture was stirred for about 30 minutes and hexane was layered. After 2 weeks, X-ray quality dark-purple flake-shaped crystals of [K(222)][Mn(T*p*ClPP)(*p*-CH_3_PhS^−^)] were obtained and a large well-formed specimen was selected for the diffraction experiment.

## Refinement

Crystal data, data collection and structure refinement details are summarized in Table 1 ▸. Anisotropic displacement parameters (ADP) of C70*A*, C71*A* and C72*A* were restrained with the RIGU instruction to get a better disorder model. The refined occupancy ratio of the disordered atoms is 0.576 (7):0.524 (7).

## Supplementary Material

Crystal structure: contains datablock(s) I. DOI: 10.1107/S2414314622002413/bt4121sup1.cif

Structure factors: contains datablock(s) I. DOI: 10.1107/S2414314622002413/bt4121Isup3.hkl

CCDC reference: 2142607

Additional supporting information:  crystallographic information; 3D view; checkCIF report

## Figures and Tables

**Table 1:** Experimental details

Crystal data	
Chemical formula	[K(C_18_H_36_N_2_O_6_)][Mn(C_44_H_24_Cl_4_N_4_)(C_7_H_7_S)]·2C_4_H_8_O
*M*_r_	1488.39
Crystal system, space group	Orthorhombic, *P* *b* *c* *a*
Temperature (K)	100
*a*, *b*, *c* (Å)	19.5585 (8), 24.7224 (10), 29.8538 (10)
*V* (Å^3^)	14435.3 (10)
*Z*	8
Radiation type	Mo *K*α
μ (mm^−1^)	0.48
Crystal size (mm)	0.50 × 0.15 × 0.07

Data collection	
Diffractometer	Bruker SMART APEX CCD
Absorption correction	Multi-scan (*SADABS*; Bruker, 2013 ▸)
*T*_min_, *T*_max_	0.643, 0.895
No. of measured, independent and observed [*I* > 2σ(*I*)] reflections	102634, 14709, 10154
*R*_int_	0.090
(sin θ/λ)_max_ (Å^−1^)	0.625

Refinement	
*R*[*F* ^2^ > 2σ(*F* ^2^)], *wR*(*F* ^2^), *S*	0.049, 0.115, 1.03
No. of reflections	14709
No. of parameters	930
No. of restraints	9
H-atom treatment	H-atom parameters constrained
Δρ_max_, Δρ_min_ (e Å^−3^)	0.58, −0.71

Computer programs: *APEX2* and *SAINT* (Bruker, 2013 ▸), *SHELXT2014/4* (Sheldrick, 2015*a* ▸), *SHELXL2019/2* (Sheldrick, 2015*b* ▸) and *OLEX2* (Dolomanov *et al.*, 2009 ▸).

## full crystallographic data

### Crystal data

**Table:**

[K(C_18_H_36_N_2_O_6_)][Mn(C_44_H_24_Cl_4_N_4_)(C_7_H_7_S)]·2C_4_H_8_O	*D*_x_ = 1.370 Mg m^−^^3^
*M**_r_* = 1488.39	Mo *K*α radiation, λ = 0.71073 Å
Orthorhombic, *P**b**c**a*	Cell parameters from 2316 reflections
*a* = 19.5585 (8) Å	θ = 3–23°
*b* = 24.7224 (10) Å	µ = 0.48 mm^−^^1^
*c* = 29.8538 (10) Å	*T* = 100 K
*V* = 14435.3 (10) Å^3^	Prism, violet
*Z* = 8	0.50 × 0.15 × 0.07 mm
*F*(000) = 6232	

### Data collection

**Table:**

Bruker SMART APEX CCD diffractometer	10154 reflections with *I* > 2σ(*I*)
ω and φ scan	*R*_int_ = 0.090
Absorption correction: multi-scan (SADABS; Bruker, 2013)	θ_max_ = 26.4°, θ_min_ = 1.9°
*T*_min_ = 0.643, *T*_max_ = 0.895	*h* = −24→24
102634 measured reflections	*k* = −24→30
14709 independent reflections	*l* = −34→37

### Refinement

**Table:**

Refinement on *F*^2^	Primary atom site location: dual
Least-squares matrix: full	Hydrogen site location: inferred from neighbouring sites
*R*[*F*^2^ > 2σ(*F*^2^)] = 0.049	H-atom parameters constrained
*wR*(*F*^2^) = 0.115	*w* = 1/[σ^2^(*F*_o_^2^) + (0.0387*P*)^2^ + 21.8473*P*] where *P* = (*F*_o_^2^ + 2*F*_c_^2^)/3
*S* = 1.03	(Δ/σ)_max_ = 0.001
14709 reflections	Δρ_max_ = 0.58 e Å^−^^3^
930 parameters	Δρ_min_ = −0.71 e Å^−^^3^
9 restraints	

### Special details

**Table:**

Geometry. All esds (except the esd in the dihedral angle between two l.s. planes) are estimated using the full covariance matrix. The cell esds are taken into account individually in the estimation of esds in distances, angles and torsion angles; correlations between esds in cell parameters are only used when they are defined by crystal symmetry. An approximate (isotropic) treatment of cell esds is used for estimating esds involving l.s. planes.
Refinement. Only one RIGU instruction was performed to get a better disorder model. H atoms were placed in calculated positions (C—H = 0.95, 0.98 or 0.99 Å for aryl, methyl or methine H atoms, respectively) and were refined using a riding model with *U*_iso_(H) = 1.5*U*_eq_(C) for methyl H atoms or *U*_iso_(H) = 1.2*U*_eq_(C, N).

### Fractional atomic coordinates and isotropic or equivalent isotropic displacement parameters (Å^2^)

**Table:**

	*x*	*y*	*z*	*U*_iso_*/*U*_eq_	Occ. (<1)
Mn1	0.41502 (2)	0.39475 (2)	0.25672 (2)	0.01269 (10)	
Cl1	0.05866 (4)	0.22569 (3)	0.08707 (3)	0.0339 (2)	
Cl2	0.29708 (5)	0.20842 (4)	0.52001 (3)	0.0439 (2)	
Cl3	0.61267 (5)	0.65068 (4)	0.46035 (3)	0.0410 (2)	
Cl4	0.57915 (5)	0.57763 (4)	0.00245 (3)	0.0444 (2)	
S1	0.50895 (4)	0.32983 (3)	0.24506 (2)	0.02333 (17)	
N1	0.32172 (11)	0.35047 (9)	0.27139 (7)	0.0130 (5)	
N2	0.41490 (11)	0.41426 (9)	0.32704 (7)	0.0153 (5)	
N3	0.45710 (11)	0.47513 (9)	0.24885 (7)	0.0139 (5)	
N6	0.36881 (11)	0.40802 (9)	0.19239 (7)	0.0135 (5)	
C1	0.27870 (13)	0.32733 (11)	0.24053 (8)	0.0133 (5)	
C2	0.23966 (13)	0.28507 (11)	0.26193 (8)	0.0151 (6)	
H2	0.206079	0.262828	0.248166	0.018*	
C3	0.25979 (13)	0.28306 (11)	0.30522 (9)	0.0161 (6)	
H3	0.243689	0.258634	0.327405	0.019*	
C4	0.31027 (13)	0.32490 (11)	0.31146 (8)	0.0130 (5)	
C5	0.34156 (13)	0.33889 (11)	0.35245 (8)	0.0143 (6)	
C6	0.38523 (14)	0.38328 (11)	0.35961 (8)	0.0162 (6)	
C7	0.40091 (14)	0.40570 (11)	0.40324 (9)	0.0195 (6)	
H7	0.387126	0.391513	0.431402	0.023*	
C8	0.43871 (15)	0.45042 (12)	0.39635 (9)	0.0219 (6)	
H8	0.455629	0.474234	0.418729	0.026*	
C9	0.44879 (14)	0.45549 (11)	0.34859 (9)	0.0166 (6)	
C10	0.48675 (14)	0.49664 (11)	0.32725 (9)	0.0161 (6)	
C11	0.49245 (14)	0.50407 (11)	0.28064 (9)	0.0152 (6)	
C12	0.53766 (14)	0.54191 (11)	0.25861 (9)	0.0174 (6)	
H12	0.567185	0.567105	0.272861	0.021*	
C13	0.53017 (14)	0.53484 (11)	0.21417 (9)	0.0175 (6)	
H13	0.554409	0.553321	0.191238	0.021*	
C14	0.47816 (14)	0.49370 (11)	0.20786 (9)	0.0151 (6)	
C15	0.45158 (13)	0.47704 (10)	0.16616 (8)	0.0135 (5)	
C16	0.39791 (13)	0.43966 (11)	0.15968 (8)	0.0134 (6)	
C17	0.36211 (14)	0.43094 (11)	0.11781 (9)	0.0165 (6)	
H17	0.372124	0.447569	0.089888	0.020*	
C18	0.31196 (14)	0.39482 (11)	0.12586 (8)	0.0160 (6)	
H18	0.279390	0.381846	0.104837	0.019*	
C19	0.31703 (13)	0.37952 (10)	0.17259 (8)	0.0130 (5)	
C20	0.27486 (13)	0.34148 (10)	0.19461 (8)	0.0122 (5)	
C21	0.22111 (13)	0.31340 (11)	0.16745 (8)	0.0142 (6)	
C22	0.23845 (14)	0.28242 (11)	0.12996 (8)	0.0159 (6)	
H22	0.285048	0.279555	0.121290	0.019*	
C23	0.18862 (14)	0.25572 (12)	0.10514 (9)	0.0190 (6)	
H23	0.200862	0.234792	0.079691	0.023*	
C24	0.12097 (14)	0.26013 (12)	0.11811 (9)	0.0203 (6)	
C25	0.10189 (14)	0.29056 (12)	0.15482 (9)	0.0210 (6)	
H25	0.055173	0.293238	0.163250	0.025*	
C26	0.15211 (14)	0.31720 (12)	0.17920 (9)	0.0191 (6)	
H26	0.139311	0.338418	0.204367	0.023*	
C27	0.32716 (14)	0.30589 (11)	0.39320 (8)	0.0154 (6)	
C28	0.38159 (14)	0.28166 (11)	0.41540 (9)	0.0187 (6)	
H28	0.426307	0.285466	0.403384	0.022*	
C29	0.37292 (16)	0.25220 (12)	0.45434 (9)	0.0239 (7)	
H29	0.410856	0.235670	0.468749	0.029*	
C30	0.30837 (17)	0.24740 (12)	0.47166 (9)	0.0262 (7)	
C31	0.25273 (17)	0.27096 (13)	0.45141 (10)	0.0298 (7)	
H31	0.208407	0.267339	0.464078	0.036*	
C32	0.26236 (15)	0.30042 (12)	0.41178 (9)	0.0236 (7)	
H32	0.224225	0.316798	0.397482	0.028*	
C33	0.52198 (14)	0.53571 (11)	0.35799 (9)	0.0172 (6)	
C34	0.50405 (14)	0.59012 (11)	0.35869 (9)	0.0187 (6)	
H34	0.471738	0.603321	0.337596	0.022*	
C35	0.53247 (15)	0.62560 (12)	0.38967 (9)	0.0226 (6)	
H35	0.520535	0.662850	0.389391	0.027*	
C36	0.57828 (15)	0.60590 (13)	0.42084 (9)	0.0266 (7)	
C37	0.59704 (16)	0.55245 (13)	0.42148 (10)	0.0278 (7)	
H37	0.628299	0.539481	0.443341	0.033*	
C38	0.56963 (15)	0.51743 (12)	0.38964 (10)	0.0237 (7)	
H38	0.583449	0.480588	0.389362	0.028*	
C39	0.48171 (13)	0.50189 (11)	0.12495 (8)	0.0152 (6)	
C40	0.4701 (2)	0.55485 (13)	0.11303 (11)	0.0389 (9)	
H40	0.441518	0.576502	0.131453	0.047*	
C41	0.49884 (18)	0.57764 (13)	0.07509 (10)	0.0348 (8)	
H41	0.489334	0.614192	0.067494	0.042*	
C42	0.54061 (14)	0.54744 (13)	0.04883 (9)	0.0229 (7)	
C43	0.5522 (2)	0.49456 (17)	0.05898 (14)	0.0679 (15)	
H43	0.580022	0.473005	0.039965	0.082*	
C44	0.5232 (2)	0.47218 (15)	0.09725 (12)	0.0485 (11)	
H44	0.532381	0.435436	0.104386	0.058*	
C45	0.56731 (15)	0.33418 (13)	0.16100 (10)	0.0267 (7)	
H45	0.605423	0.351118	0.174870	0.032*	
C46	0.56887 (16)	0.32318 (13)	0.11505 (11)	0.0304 (7)	
H46	0.608390	0.332836	0.098364	0.036*	
C47	0.51442 (16)	0.29853 (13)	0.09293 (10)	0.0288 (7)	
C48	0.45680 (16)	0.28586 (12)	0.11821 (10)	0.0265 (7)	
H48	0.418509	0.269484	0.104081	0.032*	
C49	0.45454 (15)	0.29688 (12)	0.16391 (10)	0.0239 (7)	
H49	0.414338	0.288242	0.180276	0.029*	
C50	0.51032 (15)	0.32051 (12)	0.18660 (10)	0.0236 (7)	
C51	0.51692 (18)	0.28690 (16)	0.04327 (11)	0.0425 (9)	
H51A	0.490606	0.314433	0.027124	0.064*	
H51B	0.497255	0.251128	0.037434	0.064*	
H51C	0.564520	0.287599	0.033028	0.064*	
K1	0.25036 (3)	0.10692 (3)	0.28299 (2)	0.01882 (13)	
O1	0.35824 (9)	0.15748 (7)	0.23211 (6)	0.0190 (4)	
O2	0.35312 (9)	0.16751 (8)	0.32568 (6)	0.0212 (4)	
O3	0.26008 (11)	0.00865 (8)	0.23742 (6)	0.0264 (5)	
O4	0.27592 (11)	0.00809 (8)	0.33040 (7)	0.0281 (5)	
O5	0.14182 (9)	0.16472 (8)	0.23977 (6)	0.0220 (4)	
O6	0.13926 (10)	0.15556 (8)	0.33273 (6)	0.0229 (5)	
N4	0.24340 (12)	0.10634 (10)	0.18324 (7)	0.0205 (5)	
N5	0.25364 (13)	0.10717 (10)	0.38331 (8)	0.0246 (6)	
C52	0.30792 (15)	0.12625 (12)	0.16406 (9)	0.0217 (6)	
H52A	0.300010	0.136540	0.132437	0.026*	
H52B	0.341849	0.096532	0.164365	0.026*	
C53	0.33718 (15)	0.17405 (12)	0.18868 (9)	0.0204 (6)	
H53A	0.376759	0.188735	0.171960	0.024*	
H53B	0.302246	0.202867	0.191161	0.024*	
C54	0.39241 (14)	0.20015 (11)	0.25531 (10)	0.0198 (6)	
H54A	0.361635	0.231765	0.258086	0.024*	
H54B	0.433460	0.211444	0.238310	0.024*	
C55	0.41285 (14)	0.18086 (12)	0.30077 (10)	0.0218 (6)	
H55A	0.442620	0.148644	0.298022	0.026*	
H55B	0.438812	0.209527	0.316477	0.026*	
C56	0.36933 (15)	0.14598 (13)	0.36872 (9)	0.0241 (7)	
H56A	0.405565	0.168061	0.382931	0.029*	
H56B	0.386509	0.108506	0.365585	0.029*	
C57	0.30613 (15)	0.14643 (12)	0.39747 (9)	0.0224 (6)	
H57A	0.319381	0.138475	0.428793	0.027*	
H57B	0.286036	0.183174	0.396861	0.027*	
C58	0.22999 (16)	0.05034 (12)	0.16858 (10)	0.0272 (7)	
H58A	0.236666	0.048121	0.135759	0.033*	
H58B	0.181602	0.041419	0.174934	0.033*	
C59	0.27509 (17)	0.00871 (12)	0.19091 (10)	0.0274 (7)	
H59A	0.266269	−0.027520	0.178021	0.033*	
H59B	0.323827	0.017788	0.186038	0.033*	
C60	0.29232 (17)	−0.03531 (12)	0.26026 (10)	0.0288 (7)	
H60A	0.342056	−0.028608	0.262905	0.035*	
H60B	0.285438	−0.069368	0.243409	0.035*	
C61	0.26097 (18)	−0.03967 (12)	0.30558 (10)	0.0328 (8)	
H61A	0.210845	−0.044141	0.302801	0.039*	
H61B	0.279521	−0.071674	0.321375	0.039*	
C62	0.2424 (2)	0.00788 (14)	0.37273 (10)	0.0392 (9)	
H62A	0.249704	−0.027238	0.387943	0.047*	
H62B	0.192648	0.013318	0.368703	0.047*	
C63	0.27203 (19)	0.05307 (13)	0.40036 (10)	0.0365 (8)	
H63A	0.255569	0.049533	0.431586	0.044*	
H63B	0.322455	0.049594	0.400749	0.044*	
C64	0.18716 (15)	0.14145 (13)	0.16860 (9)	0.0254 (7)	
H64A	0.173617	0.130782	0.137897	0.031*	
H64B	0.204073	0.179153	0.167227	0.031*	
C65	0.12488 (15)	0.13991 (14)	0.19810 (10)	0.0269 (7)	
H65A	0.086742	0.159634	0.183658	0.032*	
H65B	0.110411	0.102009	0.203053	0.032*	
C66	0.08185 (14)	0.17733 (13)	0.26481 (10)	0.0243 (7)	
H66A	0.052856	0.144701	0.267745	0.029*	
H66B	0.055128	0.205535	0.249063	0.029*	
C67	0.10215 (14)	0.19717 (12)	0.31016 (10)	0.0236 (7)	
H67A	0.131014	0.229873	0.307238	0.028*	
H67B	0.060891	0.206843	0.327619	0.028*	
C68	0.15332 (15)	0.16976 (14)	0.37793 (9)	0.0281 (7)	
H68A	0.110324	0.179679	0.393383	0.034*	
H68B	0.184400	0.201343	0.378832	0.034*	
C69	0.18590 (16)	0.12271 (14)	0.40108 (10)	0.0313 (8)	
H69A	0.190805	0.131527	0.433269	0.038*	
H69B	0.154901	0.091180	0.398728	0.038*	
O7	0.1968 (4)	0.4155 (3)	0.4604 (2)	0.0480 (19)	0.576 (7)
C70	0.2086 (5)	0.4307 (4)	0.5387 (3)	0.044 (2)	0.576 (7)
H70A	0.250667	0.428321	0.556998	0.053*	0.576 (7)
H70B	0.172995	0.450068	0.555996	0.053*	0.576 (7)
C71	0.2225 (4)	0.4589 (3)	0.4947 (3)	0.0432 (19)	0.576 (7)
H71A	0.271815	0.466566	0.490748	0.052*	0.576 (7)
H71B	0.196327	0.493025	0.492110	0.052*	0.576 (7)
C72	0.1554 (4)	0.3800 (4)	0.4822 (3)	0.024 (2)	0.576 (7)
H72A	0.154776	0.344582	0.466778	0.028*	0.576 (7)
H72B	0.108100	0.394059	0.484020	0.028*	0.576 (7)
C73	0.1842 (4)	0.3753 (3)	0.5250 (2)	0.045 (2)	0.576 (7)
H73A	0.222963	0.349539	0.524421	0.054*	0.576 (7)
H73B	0.149644	0.361775	0.546511	0.054*	0.576 (7)
O7A	0.1532 (5)	0.4369 (5)	0.5368 (3)	0.096 (4)	0.424 (7)
C70A	0.1391 (6)	0.3923 (5)	0.4998 (4)	0.039 (3)	0.424 (7)
H70C	0.147630	0.356229	0.513080	0.047*	0.424 (7)
H70D	0.090231	0.393918	0.491273	0.047*	0.424 (7)
C71A	0.1717 (7)	0.3966 (7)	0.4681 (5)	0.038 (3)	0.424 (7)
H71C	0.149567	0.421411	0.446459	0.046*	0.424 (7)
H71D	0.178248	0.360981	0.453514	0.046*	0.424 (7)
C72A	0.2445 (4)	0.4211 (5)	0.4848 (3)	0.038 (3)	0.424 (7)
H72C	0.276073	0.391735	0.493840	0.045*	0.424 (7)
H72D	0.266248	0.442868	0.460972	0.045*	0.424 (7)
C73A	0.2258 (7)	0.4555 (8)	0.5240 (5)	0.079 (6)	0.424 (7)
H73C	0.226139	0.494301	0.515731	0.095*	0.424 (7)
H73D	0.258062	0.449771	0.549123	0.095*	0.424 (7)
O8	0.63548 (14)	0.45376 (10)	0.49776 (9)	0.0489 (7)	
C74	0.56899 (19)	0.43233 (17)	0.50538 (12)	0.0462 (10)	
H74A	0.534356	0.454053	0.489218	0.055*	
H74B	0.558080	0.433138	0.537755	0.055*	
C75	0.5688 (3)	0.37536 (19)	0.48859 (16)	0.0695 (14)	
H75A	0.523462	0.365058	0.476549	0.083*	
H75B	0.582171	0.349533	0.512364	0.083*	
C76	0.6217 (3)	0.3782 (2)	0.45221 (18)	0.0816 (16)	
H76A	0.645132	0.342960	0.448703	0.098*	
H76B	0.600500	0.388263	0.423268	0.098*	
C77	0.6703 (2)	0.42032 (18)	0.46707 (13)	0.0562 (11)	
H77A	0.710503	0.403484	0.481582	0.067*	
H77B	0.686231	0.441825	0.441124	0.067*	

### Atomic displacement parameters (Å^2^)

**Table:**

	*U*^11^	*U*^22^	*U*^33^	*U*^12^	*U*^13^	*U*^23^
Mn1	0.0146 (2)	0.0129 (2)	0.01061 (19)	−0.00261 (17)	−0.00094 (16)	0.00044 (16)
Cl1	0.0237 (4)	0.0393 (5)	0.0388 (4)	−0.0088 (4)	−0.0120 (3)	−0.0092 (4)
Cl2	0.0788 (7)	0.0359 (5)	0.0170 (4)	−0.0093 (5)	0.0140 (4)	0.0061 (3)
Cl3	0.0461 (5)	0.0431 (5)	0.0338 (4)	−0.0125 (4)	−0.0108 (4)	−0.0170 (4)
Cl4	0.0433 (5)	0.0523 (6)	0.0375 (5)	−0.0001 (4)	0.0207 (4)	0.0225 (4)
S1	0.0220 (4)	0.0272 (4)	0.0207 (4)	0.0070 (3)	−0.0046 (3)	−0.0005 (3)
N1	0.0138 (11)	0.0134 (12)	0.0117 (11)	−0.0004 (9)	0.0009 (9)	−0.0009 (9)
N2	0.0155 (12)	0.0171 (12)	0.0131 (11)	−0.0027 (10)	−0.0014 (9)	−0.0017 (9)
N3	0.0167 (12)	0.0124 (11)	0.0126 (11)	−0.0013 (9)	−0.0024 (9)	−0.0002 (9)
N6	0.0138 (12)	0.0126 (12)	0.0141 (11)	−0.0024 (9)	0.0012 (9)	−0.0001 (9)
C1	0.0112 (13)	0.0143 (14)	0.0146 (13)	0.0018 (11)	0.0008 (10)	−0.0014 (11)
C2	0.0114 (13)	0.0175 (14)	0.0165 (13)	−0.0017 (11)	0.0000 (11)	−0.0019 (11)
C3	0.0133 (14)	0.0178 (15)	0.0171 (13)	−0.0006 (11)	0.0024 (11)	0.0015 (11)
C4	0.0130 (13)	0.0140 (14)	0.0120 (13)	0.0013 (11)	0.0028 (10)	0.0002 (11)
C5	0.0144 (14)	0.0156 (14)	0.0128 (13)	0.0017 (11)	0.0012 (10)	0.0005 (11)
C6	0.0187 (14)	0.0180 (15)	0.0118 (13)	0.0008 (12)	−0.0005 (11)	−0.0002 (11)
C7	0.0262 (16)	0.0215 (16)	0.0107 (13)	−0.0032 (12)	0.0001 (11)	0.0005 (11)
C8	0.0273 (16)	0.0223 (16)	0.0161 (14)	−0.0047 (13)	−0.0062 (12)	−0.0025 (12)
C9	0.0178 (14)	0.0179 (15)	0.0142 (13)	−0.0019 (12)	−0.0027 (11)	−0.0014 (11)
C10	0.0179 (14)	0.0135 (14)	0.0169 (13)	−0.0008 (11)	−0.0049 (11)	−0.0014 (11)
C11	0.0159 (14)	0.0129 (14)	0.0168 (13)	−0.0006 (11)	−0.0031 (11)	−0.0002 (11)
C12	0.0201 (15)	0.0126 (14)	0.0193 (14)	−0.0026 (11)	−0.0050 (12)	0.0012 (11)
C13	0.0185 (15)	0.0139 (14)	0.0200 (14)	−0.0050 (11)	−0.0007 (11)	0.0033 (11)
C14	0.0156 (14)	0.0121 (14)	0.0176 (13)	0.0030 (11)	−0.0002 (11)	0.0008 (11)
C15	0.0164 (14)	0.0098 (13)	0.0142 (13)	0.0017 (11)	0.0019 (11)	0.0014 (10)
C16	0.0155 (14)	0.0123 (13)	0.0122 (13)	0.0020 (11)	0.0022 (10)	−0.0003 (10)
C17	0.0200 (15)	0.0173 (15)	0.0122 (13)	0.0001 (12)	0.0013 (11)	0.0013 (11)
C18	0.0175 (14)	0.0172 (14)	0.0135 (13)	−0.0009 (12)	−0.0033 (11)	0.0005 (11)
C19	0.0128 (13)	0.0125 (13)	0.0136 (13)	0.0026 (11)	0.0000 (10)	−0.0022 (10)
C20	0.0099 (13)	0.0122 (13)	0.0147 (13)	0.0012 (10)	0.0004 (10)	−0.0011 (11)
C21	0.0147 (14)	0.0157 (14)	0.0124 (13)	−0.0026 (11)	−0.0019 (11)	0.0035 (11)
C22	0.0148 (14)	0.0176 (15)	0.0152 (13)	−0.0025 (11)	0.0003 (11)	0.0024 (11)
C23	0.0231 (16)	0.0198 (15)	0.0140 (13)	−0.0021 (12)	−0.0009 (12)	−0.0021 (12)
C24	0.0176 (15)	0.0218 (16)	0.0215 (15)	−0.0070 (12)	−0.0063 (12)	0.0029 (12)
C25	0.0110 (14)	0.0283 (17)	0.0238 (15)	−0.0016 (12)	−0.0006 (11)	0.0038 (13)
C26	0.0182 (15)	0.0232 (16)	0.0158 (14)	−0.0011 (12)	0.0025 (11)	−0.0001 (12)
C27	0.0192 (15)	0.0154 (14)	0.0117 (13)	−0.0038 (11)	0.0014 (11)	−0.0027 (11)
C28	0.0207 (15)	0.0196 (15)	0.0159 (13)	−0.0060 (12)	−0.0017 (12)	0.0011 (12)
C29	0.0346 (18)	0.0227 (16)	0.0144 (14)	−0.0053 (14)	−0.0052 (13)	0.0015 (12)
C30	0.047 (2)	0.0208 (16)	0.0112 (14)	−0.0055 (15)	0.0061 (13)	−0.0005 (12)
C31	0.0341 (18)	0.0293 (18)	0.0260 (16)	−0.0069 (15)	0.0199 (14)	−0.0046 (14)
C32	0.0230 (16)	0.0241 (16)	0.0235 (15)	−0.0003 (13)	0.0069 (12)	−0.0005 (13)
C33	0.0187 (15)	0.0192 (15)	0.0139 (13)	−0.0064 (12)	−0.0008 (11)	0.0001 (11)
C34	0.0180 (15)	0.0212 (16)	0.0168 (13)	0.0004 (12)	−0.0019 (11)	−0.0011 (12)
C35	0.0225 (16)	0.0195 (16)	0.0258 (16)	−0.0018 (12)	0.0021 (13)	−0.0048 (13)
C36	0.0269 (17)	0.0330 (18)	0.0200 (15)	−0.0120 (14)	−0.0027 (13)	−0.0085 (14)
C37	0.0288 (18)	0.0311 (18)	0.0236 (16)	−0.0076 (14)	−0.0124 (13)	0.0031 (13)
C38	0.0258 (17)	0.0180 (15)	0.0272 (16)	−0.0028 (13)	−0.0105 (13)	0.0037 (13)
C39	0.0118 (14)	0.0199 (15)	0.0140 (13)	−0.0057 (11)	−0.0017 (10)	0.0004 (11)
C40	0.068 (3)	0.0216 (18)	0.0272 (17)	0.0132 (17)	0.0250 (17)	0.0054 (14)
C41	0.059 (2)	0.0187 (17)	0.0265 (17)	0.0002 (16)	0.0122 (16)	0.0067 (13)
C42	0.0185 (15)	0.0316 (18)	0.0185 (15)	−0.0080 (13)	0.0005 (12)	0.0087 (13)
C43	0.084 (3)	0.050 (3)	0.070 (3)	0.035 (2)	0.066 (3)	0.030 (2)
C44	0.061 (3)	0.030 (2)	0.054 (2)	0.0221 (18)	0.039 (2)	0.0225 (17)
C45	0.0210 (16)	0.0277 (17)	0.0315 (17)	0.0010 (13)	−0.0051 (13)	0.0012 (14)
C46	0.0242 (17)	0.0320 (19)	0.0350 (18)	0.0047 (14)	0.0062 (14)	0.0053 (15)
C47	0.0272 (17)	0.0314 (18)	0.0277 (16)	0.0076 (14)	−0.0007 (14)	−0.0004 (14)
C48	0.0229 (17)	0.0261 (18)	0.0306 (17)	0.0019 (13)	−0.0028 (13)	−0.0004 (14)
C49	0.0207 (16)	0.0220 (16)	0.0290 (16)	0.0038 (13)	−0.0002 (13)	0.0011 (13)
C50	0.0251 (16)	0.0197 (16)	0.0260 (15)	0.0082 (13)	−0.0011 (13)	−0.0007 (13)
C51	0.040 (2)	0.061 (3)	0.0270 (18)	−0.0004 (19)	0.0010 (15)	−0.0080 (17)
K1	0.0179 (3)	0.0198 (3)	0.0188 (3)	−0.0032 (3)	−0.0027 (2)	0.0012 (3)
O1	0.0188 (10)	0.0145 (10)	0.0239 (10)	−0.0024 (8)	−0.0022 (8)	−0.0016 (8)
O2	0.0138 (10)	0.0282 (12)	0.0216 (10)	0.0005 (9)	−0.0035 (8)	−0.0017 (9)
O3	0.0371 (13)	0.0183 (11)	0.0238 (11)	−0.0040 (9)	−0.0008 (9)	−0.0002 (9)
O4	0.0413 (13)	0.0181 (11)	0.0248 (11)	−0.0103 (10)	−0.0055 (9)	0.0016 (9)
O5	0.0139 (10)	0.0288 (12)	0.0231 (10)	−0.0034 (9)	−0.0026 (8)	0.0006 (9)
O6	0.0205 (11)	0.0272 (12)	0.0210 (10)	−0.0009 (9)	−0.0012 (8)	0.0009 (9)
N4	0.0212 (13)	0.0202 (13)	0.0203 (12)	−0.0054 (11)	−0.0022 (10)	0.0011 (10)
N5	0.0302 (14)	0.0227 (14)	0.0210 (12)	−0.0007 (11)	−0.0049 (11)	0.0025 (11)
C52	0.0239 (16)	0.0223 (16)	0.0189 (15)	−0.0028 (13)	0.0021 (12)	0.0005 (12)
C53	0.0197 (15)	0.0195 (15)	0.0220 (15)	−0.0020 (12)	0.0039 (12)	0.0023 (12)
C54	0.0110 (13)	0.0164 (14)	0.0320 (16)	−0.0020 (11)	0.0015 (12)	−0.0036 (13)
C55	0.0136 (14)	0.0191 (15)	0.0328 (16)	−0.0013 (12)	−0.0019 (12)	−0.0069 (13)
C56	0.0225 (16)	0.0254 (17)	0.0245 (16)	0.0065 (13)	−0.0088 (12)	−0.0057 (13)
C57	0.0244 (16)	0.0246 (17)	0.0182 (14)	0.0033 (13)	−0.0061 (12)	−0.0023 (12)
C58	0.0337 (18)	0.0240 (17)	0.0239 (16)	−0.0123 (14)	−0.0044 (13)	−0.0029 (13)
C59	0.0378 (19)	0.0193 (16)	0.0251 (16)	−0.0086 (14)	0.0014 (14)	−0.0037 (13)
C60	0.0392 (19)	0.0134 (15)	0.0337 (18)	−0.0060 (14)	−0.0017 (15)	−0.0009 (13)
C61	0.050 (2)	0.0150 (16)	0.0330 (18)	−0.0113 (15)	−0.0059 (16)	0.0037 (13)
C62	0.064 (3)	0.0291 (19)	0.0241 (17)	−0.0124 (18)	−0.0019 (16)	0.0071 (14)
C63	0.060 (2)	0.0269 (19)	0.0225 (16)	−0.0015 (17)	−0.0110 (16)	0.0045 (14)
C64	0.0252 (16)	0.0307 (18)	0.0204 (15)	−0.0063 (14)	−0.0055 (12)	0.0035 (13)
C65	0.0211 (16)	0.0361 (19)	0.0236 (16)	−0.0070 (14)	−0.0073 (13)	0.0036 (14)
C66	0.0136 (14)	0.0295 (17)	0.0299 (16)	0.0003 (13)	0.0003 (13)	0.0080 (13)
C67	0.0152 (15)	0.0214 (16)	0.0341 (17)	0.0005 (12)	0.0036 (13)	0.0052 (13)
C68	0.0191 (16)	0.045 (2)	0.0205 (15)	−0.0011 (14)	0.0052 (12)	−0.0028 (14)
C69	0.0305 (18)	0.043 (2)	0.0204 (16)	−0.0080 (15)	0.0020 (13)	0.0020 (14)
O7	0.054 (5)	0.045 (4)	0.045 (3)	0.006 (3)	0.000 (3)	−0.007 (3)
C70	0.033 (5)	0.065 (6)	0.035 (5)	−0.004 (4)	0.002 (4)	−0.006 (4)
C71	0.047 (4)	0.035 (4)	0.047 (5)	−0.004 (3)	0.006 (4)	0.007 (4)
C72	0.030 (5)	0.016 (4)	0.025 (6)	−0.007 (3)	0.004 (4)	0.001 (3)
C73	0.050 (4)	0.044 (4)	0.041 (4)	−0.005 (3)	0.007 (3)	0.013 (3)
O7A	0.074 (7)	0.153 (10)	0.061 (5)	−0.030 (6)	0.015 (5)	−0.009 (5)
C70A	0.031 (5)	0.043 (7)	0.043 (7)	0.006 (4)	−0.020 (4)	−0.009 (5)
C71A	0.031 (7)	0.052 (11)	0.032 (7)	0.017 (5)	−0.022 (5)	−0.013 (6)
C72A	0.025 (4)	0.069 (7)	0.020 (4)	0.008 (4)	−0.008 (3)	0.004 (4)
C73A	0.046 (8)	0.150 (17)	0.042 (8)	−0.032 (9)	0.027 (6)	−0.008 (10)
O8	0.0583 (17)	0.0364 (15)	0.0520 (16)	0.0000 (13)	0.0090 (13)	−0.0034 (12)
C74	0.041 (2)	0.059 (3)	0.038 (2)	0.0053 (19)	0.0001 (17)	−0.0007 (19)
C75	0.074 (3)	0.060 (3)	0.075 (3)	−0.022 (3)	−0.018 (3)	0.006 (3)
C76	0.118 (5)	0.046 (3)	0.081 (4)	0.016 (3)	−0.002 (3)	−0.023 (3)
C77	0.070 (3)	0.054 (3)	0.045 (2)	0.011 (2)	0.012 (2)	−0.004 (2)

### Geometric parameters (Å, º)

**Table:**

Mn1—S1	2.4642 (8)	K1—O4	2.867 (2)
Mn1—N1	2.173 (2)	K1—O5	2.866 (2)
Mn1—N2	2.154 (2)	K1—O6	2.893 (2)
Mn1—N3	2.164 (2)	K1—N4	2.981 (2)
Mn1—N6	2.148 (2)	K1—N5	2.996 (2)
Cl1—C24	1.752 (3)	O1—C53	1.421 (3)
Cl2—C30	1.750 (3)	O1—C54	1.428 (3)
Cl3—C36	1.752 (3)	O2—C55	1.424 (3)
Cl4—C42	1.744 (3)	O2—C56	1.426 (3)
S1—C50	1.761 (3)	O3—C59	1.419 (3)
N1—C1	1.373 (3)	O3—C60	1.430 (4)
N1—C4	1.371 (3)	O4—C61	1.424 (4)
N2—C6	1.367 (3)	O4—C62	1.424 (4)
N2—C9	1.376 (3)	O5—C65	1.426 (3)
N3—C11	1.375 (3)	O5—C66	1.425 (3)
N3—C14	1.370 (3)	O6—C67	1.428 (3)
N6—C16	1.375 (3)	O6—C68	1.421 (3)
N6—C19	1.368 (3)	N4—C52	1.471 (4)
C1—C2	1.443 (4)	N4—C58	1.476 (4)
C1—C20	1.417 (4)	N4—C64	1.468 (4)
C2—H2	0.9500	N5—C57	1.475 (4)
C2—C3	1.352 (4)	N5—C63	1.476 (4)
C3—H3	0.9500	N5—C69	1.478 (4)
C3—C4	1.442 (4)	C52—H52A	0.9900
C4—C5	1.411 (4)	C52—H52B	0.9900
C5—C6	1.407 (4)	C52—C53	1.505 (4)
C5—C27	1.492 (4)	C53—H53A	0.9900
C6—C7	1.449 (4)	C53—H53B	0.9900
C7—H7	0.9500	C54—H54A	0.9900
C7—C8	1.346 (4)	C54—H54B	0.9900
C8—H8	0.9500	C54—C55	1.493 (4)
C8—C9	1.445 (4)	C55—H55A	0.9900
C9—C10	1.411 (4)	C55—H55B	0.9900
C10—C11	1.408 (4)	C56—H56A	0.9900
C10—C33	1.500 (4)	C56—H56B	0.9900
C11—C12	1.445 (4)	C56—C57	1.505 (4)
C12—H12	0.9500	C57—H57A	0.9900
C12—C13	1.346 (4)	C57—H57B	0.9900
C13—H13	0.9500	C58—H58A	0.9900
C13—C14	1.451 (4)	C58—H58B	0.9900
C14—C15	1.411 (4)	C58—C59	1.510 (4)
C15—C16	1.412 (4)	C59—H59A	0.9900
C15—C39	1.496 (4)	C59—H59B	0.9900
C16—C17	1.449 (4)	C60—H60A	0.9900
C17—H17	0.9500	C60—H60B	0.9900
C17—C18	1.348 (4)	C60—C61	1.489 (4)
C18—H18	0.9500	C61—H61A	0.9900
C18—C19	1.449 (4)	C61—H61B	0.9900
C19—C20	1.413 (4)	C62—H62A	0.9900
C20—C21	1.498 (4)	C62—H62B	0.9900
C21—C22	1.398 (4)	C62—C63	1.505 (5)
C21—C26	1.397 (4)	C63—H63A	0.9900
C22—H22	0.9500	C63—H63B	0.9900
C22—C23	1.391 (4)	C64—H64A	0.9900
C23—H23	0.9500	C64—H64B	0.9900
C23—C24	1.383 (4)	C64—C65	1.503 (4)
C24—C25	1.381 (4)	C65—H65A	0.9900
C25—H25	0.9500	C65—H65B	0.9900
C25—C26	1.389 (4)	C66—H66A	0.9900
C26—H26	0.9500	C66—H66B	0.9900
C27—C28	1.390 (4)	C66—C67	1.494 (4)
C27—C32	1.390 (4)	C67—H67A	0.9900
C28—H28	0.9500	C67—H67B	0.9900
C28—C29	1.382 (4)	C68—H68A	0.9900
C29—H29	0.9500	C68—H68B	0.9900
C29—C30	1.369 (4)	C68—C69	1.495 (4)
C30—C31	1.374 (5)	C69—H69A	0.9900
C31—H31	0.9500	C69—H69B	0.9900
C31—C32	1.402 (4)	O7—C71	1.565 (10)
C32—H32	0.9500	O7—C72	1.360 (9)
C33—C34	1.390 (4)	C70—H70A	0.9900
C33—C38	1.402 (4)	C70—H70B	0.9900
C34—H34	0.9500	C70—C71	1.511 (11)
C34—C35	1.391 (4)	C70—C73	1.509 (10)
C35—H35	0.9500	C71—H71A	0.9900
C35—C36	1.381 (4)	C71—H71B	0.9900
C36—C37	1.372 (4)	C72—H72A	0.9900
C37—H37	0.9500	C72—H72B	0.9900
C37—C38	1.393 (4)	C72—C73	1.400 (10)
C38—H38	0.9500	C73—H73A	0.9900
C39—C40	1.375 (4)	C73—H73B	0.9900
C39—C44	1.372 (4)	O7A—C70A	1.584 (14)
C40—H40	0.9500	O7A—C73A	1.542 (14)
C40—C41	1.384 (4)	C70A—H70C	0.9900
C41—H41	0.9500	C70A—H70D	0.9900
C41—C42	1.357 (4)	C70A—C71A	1.147 (18)
C42—C43	1.361 (5)	C71A—H71C	0.9900
C43—H43	0.9500	C71A—H71D	0.9900
C43—C44	1.390 (5)	C71A—C72A	1.625 (15)
C44—H44	0.9500	C72A—H72C	0.9900
C45—H45	0.9500	C72A—H72D	0.9900
C45—C46	1.399 (4)	C72A—C73A	1.492 (17)
C45—C50	1.393 (4)	C73A—H73C	0.9900
C46—H46	0.9500	C73A—H73D	0.9900
C46—C47	1.393 (4)	O8—C74	1.422 (4)
C47—C48	1.392 (4)	O8—C77	1.409 (4)
C47—C51	1.511 (4)	C74—H74A	0.9900
C48—H48	0.9500	C74—H74B	0.9900
C48—C49	1.392 (4)	C74—C75	1.495 (6)
C49—H49	0.9500	C75—H75A	0.9900
C49—C50	1.411 (4)	C75—H75B	0.9900
C51—H51A	0.9800	C75—C76	1.502 (7)
C51—H51B	0.9800	C76—H76A	0.9900
C51—H51C	0.9800	C76—H76B	0.9900
K1—O1	2.8849 (19)	C76—C77	1.478 (6)
K1—O2	2.8120 (19)	C77—H77A	0.9900
K1—O3	2.791 (2)	C77—H77B	0.9900
			
N1—Mn1—S1	109.06 (6)	C55—O2—K1	118.24 (15)
N2—Mn1—S1	106.52 (6)	C55—O2—C56	112.0 (2)
N2—Mn1—N1	85.15 (8)	C56—O2—K1	111.62 (16)
N2—Mn1—N3	84.30 (8)	C59—O3—K1	119.35 (16)
N3—Mn1—S1	107.42 (6)	C59—O3—C60	112.1 (2)
N3—Mn1—N1	143.53 (8)	C60—O3—K1	117.34 (16)
N6—Mn1—S1	106.67 (6)	C61—O4—K1	114.44 (16)
N6—Mn1—N1	84.48 (8)	C62—O4—K1	111.17 (18)
N6—Mn1—N2	146.81 (8)	C62—O4—C61	111.4 (2)
N6—Mn1—N3	85.57 (8)	C65—O5—K1	110.51 (17)
C50—S1—Mn1	103.69 (10)	C66—O5—K1	118.85 (15)
C1—N1—Mn1	126.13 (16)	C66—O5—C65	111.1 (2)
C4—N1—Mn1	123.03 (17)	C67—O6—K1	116.04 (15)
C4—N1—C1	107.1 (2)	C68—O6—K1	116.40 (16)
C6—N2—Mn1	124.62 (17)	C68—O6—C67	111.6 (2)
C6—N2—C9	106.7 (2)	C52—N4—K1	110.38 (16)
C9—N2—Mn1	128.39 (17)	C52—N4—C58	110.6 (2)
C11—N3—Mn1	126.45 (17)	C58—N4—K1	108.00 (16)
C14—N3—Mn1	121.27 (17)	C64—N4—K1	109.20 (16)
C14—N3—C11	106.9 (2)	C64—N4—C52	109.2 (2)
C16—N6—Mn1	123.23 (17)	C64—N4—C58	109.5 (2)
C19—N6—Mn1	128.26 (17)	C57—N5—K1	107.63 (16)
C19—N6—C16	107.0 (2)	C57—N5—C63	109.1 (2)
N1—C1—C2	109.2 (2)	C57—N5—C69	110.5 (2)
N1—C1—C20	125.4 (2)	C63—N5—K1	110.38 (17)
C20—C1—C2	125.4 (2)	C63—N5—C69	109.3 (3)
C1—C2—H2	126.4	C69—N5—K1	109.89 (16)
C3—C2—C1	107.2 (2)	N4—C52—H52A	108.9
C3—C2—H2	126.4	N4—C52—H52B	108.9
C2—C3—H3	126.4	N4—C52—C53	113.5 (2)
C2—C3—C4	107.2 (2)	H52A—C52—H52B	107.7
C4—C3—H3	126.4	C53—C52—H52A	108.9
N1—C4—C3	109.3 (2)	C53—C52—H52B	108.9
N1—C4—C5	124.9 (2)	O1—C53—C52	109.2 (2)
C5—C4—C3	125.8 (2)	O1—C53—H53A	109.8
C4—C5—C27	119.4 (2)	O1—C53—H53B	109.8
C6—C5—C4	125.9 (2)	C52—C53—H53A	109.8
C6—C5—C27	114.7 (2)	C52—C53—H53B	109.8
N2—C6—C5	125.9 (2)	H53A—C53—H53B	108.3
N2—C6—C7	109.6 (2)	O1—C54—H54A	109.8
C5—C6—C7	124.3 (2)	O1—C54—H54B	109.8
C6—C7—H7	126.5	O1—C54—C55	109.3 (2)
C8—C7—C6	107.0 (2)	H54A—C54—H54B	108.3
C8—C7—H7	126.5	C55—C54—H54A	109.8
C7—C8—H8	126.4	C55—C54—H54B	109.8
C7—C8—C9	107.3 (2)	O2—C55—C54	109.2 (2)
C9—C8—H8	126.4	O2—C55—H55A	109.8
N2—C9—C8	109.3 (2)	O2—C55—H55B	109.8
N2—C9—C10	125.2 (2)	C54—C55—H55A	109.8
C10—C9—C8	125.4 (2)	C54—C55—H55B	109.8
C9—C10—C33	115.5 (2)	H55A—C55—H55B	108.3
C11—C10—C9	125.6 (2)	O2—C56—H56A	109.8
C11—C10—C33	119.0 (2)	O2—C56—H56B	109.8
N3—C11—C10	125.1 (2)	O2—C56—C57	109.2 (2)
N3—C11—C12	109.3 (2)	H56A—C56—H56B	108.3
C10—C11—C12	125.6 (2)	C57—C56—H56A	109.8
C11—C12—H12	126.3	C57—C56—H56B	109.8
C13—C12—C11	107.3 (2)	N5—C57—C56	113.8 (2)
C13—C12—H12	126.3	N5—C57—H57A	108.8
C12—C13—H13	126.4	N5—C57—H57B	108.8
C12—C13—C14	107.2 (2)	C56—C57—H57A	108.8
C14—C13—H13	126.4	C56—C57—H57B	108.8
N3—C14—C13	109.2 (2)	H57A—C57—H57B	107.7
N3—C14—C15	125.4 (2)	N4—C58—H58A	108.8
C15—C14—C13	125.3 (2)	N4—C58—H58B	108.8
C14—C15—C16	125.9 (2)	N4—C58—C59	113.9 (2)
C14—C15—C39	117.4 (2)	H58A—C58—H58B	107.7
C16—C15—C39	116.7 (2)	C59—C58—H58A	108.8
N6—C16—C15	125.7 (2)	C59—C58—H58B	108.8
N6—C16—C17	109.2 (2)	O3—C59—C58	108.2 (2)
C15—C16—C17	125.1 (2)	O3—C59—H59A	110.1
C16—C17—H17	126.4	O3—C59—H59B	110.1
C18—C17—C16	107.3 (2)	C58—C59—H59A	110.1
C18—C17—H17	126.4	C58—C59—H59B	110.1
C17—C18—H18	126.4	H59A—C59—H59B	108.4
C17—C18—C19	107.1 (2)	O3—C60—H60A	110.1
C19—C18—H18	126.4	O3—C60—H60B	110.1
N6—C19—C18	109.4 (2)	O3—C60—C61	107.9 (3)
N6—C19—C20	125.0 (2)	H60A—C60—H60B	108.4
C20—C19—C18	125.6 (2)	C61—C60—H60A	110.1
C1—C20—C21	116.5 (2)	C61—C60—H60B	110.1
C19—C20—C1	125.7 (2)	O4—C61—C60	109.2 (2)
C19—C20—C21	117.8 (2)	O4—C61—H61A	109.8
C22—C21—C20	121.1 (2)	O4—C61—H61B	109.8
C26—C21—C20	120.7 (2)	C60—C61—H61A	109.8
C26—C21—C22	118.2 (2)	C60—C61—H61B	109.8
C21—C22—H22	119.5	H61A—C61—H61B	108.3
C23—C22—C21	121.1 (2)	O4—C62—H62A	110.1
C23—C22—H22	119.5	O4—C62—H62B	110.1
C22—C23—H23	120.5	O4—C62—C63	107.9 (3)
C24—C23—C22	118.9 (3)	H62A—C62—H62B	108.4
C24—C23—H23	120.5	C63—C62—H62A	110.1
C23—C24—Cl1	118.6 (2)	C63—C62—H62B	110.1
C25—C24—Cl1	119.8 (2)	N5—C63—C62	113.0 (3)
C25—C24—C23	121.6 (3)	N5—C63—H63A	109.0
C24—C25—H25	120.6	N5—C63—H63B	109.0
C24—C25—C26	118.9 (3)	C62—C63—H63A	109.0
C26—C25—H25	120.6	C62—C63—H63B	109.0
C21—C26—H26	119.3	H63A—C63—H63B	107.8
C25—C26—C21	121.3 (3)	N4—C64—H64A	108.6
C25—C26—H26	119.3	N4—C64—H64B	108.6
C28—C27—C5	118.7 (2)	N4—C64—C65	114.7 (2)
C28—C27—C32	117.8 (2)	H64A—C64—H64B	107.6
C32—C27—C5	123.4 (3)	C65—C64—H64A	108.6
C27—C28—H28	118.9	C65—C64—H64B	108.6
C29—C28—C27	122.3 (3)	O5—C65—C64	108.2 (2)
C29—C28—H28	118.9	O5—C65—H65A	110.1
C28—C29—H29	120.8	O5—C65—H65B	110.1
C30—C29—C28	118.4 (3)	C64—C65—H65A	110.1
C30—C29—H29	120.8	C64—C65—H65B	110.1
C29—C30—Cl2	118.4 (2)	H65A—C65—H65B	108.4
C29—C30—C31	121.8 (3)	O5—C66—H66A	109.8
C31—C30—Cl2	119.8 (2)	O5—C66—H66B	109.8
C30—C31—H31	120.5	O5—C66—C67	109.2 (2)
C30—C31—C32	119.0 (3)	H66A—C66—H66B	108.3
C32—C31—H31	120.5	C67—C66—H66A	109.8
C27—C32—C31	120.7 (3)	C67—C66—H66B	109.8
C27—C32—H32	119.7	O6—C67—C66	109.0 (2)
C31—C32—H32	119.7	O6—C67—H67A	109.9
C34—C33—C10	121.1 (2)	O6—C67—H67B	109.9
C34—C33—C38	118.0 (3)	C66—C67—H67A	109.9
C38—C33—C10	120.7 (3)	C66—C67—H67B	109.9
C33—C34—H34	119.3	H67A—C67—H67B	108.3
C33—C34—C35	121.3 (3)	O6—C68—H68A	109.8
C35—C34—H34	119.3	O6—C68—H68B	109.8
C34—C35—H35	120.5	O6—C68—C69	109.2 (3)
C36—C35—C34	119.0 (3)	H68A—C68—H68B	108.3
C36—C35—H35	120.5	C69—C68—H68A	109.8
C35—C36—Cl3	118.7 (2)	C69—C68—H68B	109.8
C37—C36—Cl3	119.8 (2)	N5—C69—C68	114.7 (2)
C37—C36—C35	121.5 (3)	N5—C69—H69A	108.6
C36—C37—H37	120.4	N5—C69—H69B	108.6
C36—C37—C38	119.1 (3)	C68—C69—H69A	108.6
C38—C37—H37	120.4	C68—C69—H69B	108.6
C33—C38—H38	119.5	H69A—C69—H69B	107.6
C37—C38—C33	121.0 (3)	C72—O7—C71	108.6 (7)
C37—C38—H38	119.5	H70A—C70—H70B	109.0
C40—C39—C15	122.6 (3)	C71—C70—H70A	111.0
C44—C39—C15	120.6 (3)	C71—C70—H70B	111.0
C44—C39—C40	116.8 (3)	C73—C70—H70A	111.0
C39—C40—H40	118.9	C73—C70—H70B	111.0
C39—C40—C41	122.2 (3)	C73—C70—C71	103.9 (6)
C41—C40—H40	118.9	O7—C71—H71A	111.5
C40—C41—H41	120.2	O7—C71—H71B	111.5
C42—C41—C40	119.6 (3)	C70—C71—O7	101.3 (5)
C42—C41—H41	120.2	C70—C71—H71A	111.5
C41—C42—Cl4	118.9 (2)	C70—C71—H71B	111.5
C41—C42—C43	120.0 (3)	H71A—C71—H71B	109.3
C43—C42—Cl4	121.1 (2)	O7—C72—H72A	110.8
C42—C43—H43	120.1	O7—C72—H72B	110.8
C42—C43—C44	119.8 (3)	O7—C72—C73	104.6 (7)
C44—C43—H43	120.1	H72A—C72—H72B	108.9
C39—C44—C43	121.6 (3)	C73—C72—H72A	110.8
C39—C44—H44	119.2	C73—C72—H72B	110.8
C43—C44—H44	119.2	C70—C73—H73A	110.2
C46—C45—H45	119.7	C70—C73—H73B	110.2
C50—C45—H45	119.7	C72—C73—C70	107.3 (6)
C50—C45—C46	120.5 (3)	C72—C73—H73A	110.2
C45—C46—H46	118.9	C72—C73—H73B	110.2
C47—C46—C45	122.2 (3)	H73A—C73—H73B	108.5
C47—C46—H46	118.9	C73A—O7A—C70A	101.3 (9)
C46—C47—C51	121.6 (3)	O7A—C70A—H70C	108.6
C48—C47—C46	117.4 (3)	O7A—C70A—H70D	108.6
C48—C47—C51	121.0 (3)	H70C—C70A—H70D	107.6
C47—C48—H48	119.6	C71A—C70A—O7A	114.5 (12)
C49—C48—C47	120.9 (3)	C71A—C70A—H70C	108.6
C49—C48—H48	119.6	C71A—C70A—H70D	108.6
C48—C49—H49	119.1	C70A—C71A—H71C	110.7
C48—C49—C50	121.8 (3)	C70A—C71A—H71D	110.7
C50—C49—H49	119.1	C70A—C71A—C72A	105.4 (11)
C45—C50—S1	121.6 (2)	H71C—C71A—H71D	108.8
C45—C50—C49	117.1 (3)	C72A—C71A—H71C	110.7
C49—C50—S1	121.2 (2)	C72A—C71A—H71D	110.7
C47—C51—H51A	109.5	C71A—C72A—H72C	111.0
C47—C51—H51B	109.5	C71A—C72A—H72D	111.0
C47—C51—H51C	109.5	H72C—C72A—H72D	109.0
H51A—C51—H51B	109.5	C73A—C72A—C71A	103.9 (9)
H51A—C51—H51C	109.5	C73A—C72A—H72C	111.0
H51B—C51—H51C	109.5	C73A—C72A—H72D	111.0
O1—K1—O6	129.74 (6)	O7A—C73A—H73C	110.9
O1—K1—N4	60.62 (6)	O7A—C73A—H73D	110.9
O1—K1—N5	120.66 (6)	C72A—C73A—O7A	104.4 (11)
O2—K1—O1	59.01 (5)	C72A—C73A—H73C	110.9
O2—K1—O4	96.06 (6)	C72A—C73A—H73D	110.9
O2—K1—O5	117.90 (6)	H73C—C73A—H73D	108.9
O2—K1—O6	94.75 (6)	C77—O8—C74	109.1 (3)
O2—K1—N4	119.20 (6)	O8—C74—H74A	110.2
O2—K1—N5	62.00 (6)	O8—C74—H74B	110.2
O3—K1—O1	94.05 (6)	O8—C74—C75	107.4 (3)
O3—K1—O2	129.49 (6)	H74A—C74—H74B	108.5
O3—K1—O4	59.14 (6)	C75—C74—H74A	110.2
O3—K1—O5	105.37 (6)	C75—C74—H74B	110.2
O3—K1—O6	131.54 (6)	C74—C75—H75A	111.5
O3—K1—N4	60.79 (6)	C74—C75—H75B	111.5
O3—K1—N5	119.19 (7)	C74—C75—C76	101.3 (4)
O4—K1—O1	120.10 (6)	H75A—C75—H75B	109.3
O4—K1—O6	103.41 (6)	C76—C75—H75A	111.5
O4—K1—N4	119.79 (6)	C76—C75—H75B	111.5
O4—K1—N5	60.30 (6)	C75—C76—H76A	110.7
O5—K1—O1	95.08 (6)	C75—C76—H76B	110.7
O5—K1—O4	140.92 (6)	H76A—C76—H76B	108.8
O5—K1—O6	57.83 (5)	C77—C76—C75	105.0 (4)
O5—K1—N4	61.24 (6)	C77—C76—H76A	110.7
O5—K1—N5	117.71 (7)	C77—C76—H76B	110.7
O6—K1—N4	118.71 (6)	O8—C77—C76	107.4 (4)
O6—K1—N5	60.15 (6)	O8—C77—H77A	110.2
N4—K1—N5	178.60 (7)	O8—C77—H77B	110.2
C53—O1—K1	113.17 (15)	C76—C77—H77A	110.2
C53—O1—C54	111.4 (2)	C76—C77—H77B	110.2
C54—O1—K1	114.01 (15)	H77A—C77—H77B	108.5
			
Mn1—S1—C50—C45	118.3 (2)	C22—C21—C26—C25	0.7 (4)
Mn1—S1—C50—C49	−64.2 (2)	C22—C23—C24—Cl1	−179.2 (2)
Mn1—N1—C1—C2	157.46 (17)	C22—C23—C24—C25	0.4 (4)
Mn1—N1—C1—C20	−20.6 (4)	C23—C24—C25—C26	−0.1 (4)
Mn1—N1—C4—C3	−157.41 (17)	C24—C25—C26—C21	−0.4 (4)
Mn1—N1—C4—C5	24.5 (4)	C26—C21—C22—C23	−0.5 (4)
Mn1—N2—C6—C5	−10.4 (4)	C27—C5—C6—N2	168.8 (3)
Mn1—N2—C6—C7	174.27 (18)	C27—C5—C6—C7	−16.6 (4)
Mn1—N2—C9—C8	−175.05 (18)	C27—C28—C29—C30	−0.7 (4)
Mn1—N2—C9—C10	5.7 (4)	C28—C27—C32—C31	−0.5 (4)
Mn1—N3—C11—C10	−24.6 (4)	C28—C29—C30—Cl2	178.1 (2)
Mn1—N3—C11—C12	153.63 (18)	C28—C29—C30—C31	0.0 (4)
Mn1—N3—C14—C13	−154.00 (18)	C29—C30—C31—C32	0.4 (5)
Mn1—N3—C14—C15	28.3 (3)	C30—C31—C32—C27	−0.1 (5)
Mn1—N6—C16—C15	−15.5 (4)	C32—C27—C28—C29	0.9 (4)
Mn1—N6—C16—C17	167.78 (17)	C33—C10—C11—N3	−172.6 (2)
Mn1—N6—C19—C18	−167.84 (17)	C33—C10—C11—C12	9.5 (4)
Mn1—N6—C19—C20	12.7 (4)	C33—C34—C35—C36	−1.4 (4)
Cl1—C24—C25—C26	179.5 (2)	C34—C33—C38—C37	1.4 (4)
Cl2—C30—C31—C32	−177.7 (2)	C34—C35—C36—Cl3	−178.9 (2)
Cl3—C36—C37—C38	−179.5 (2)	C34—C35—C36—C37	1.0 (4)
Cl4—C42—C43—C44	177.2 (4)	C35—C36—C37—C38	0.6 (5)
N1—C1—C2—C3	−0.3 (3)	C36—C37—C38—C33	−1.8 (5)
N1—C1—C20—C19	3.6 (4)	C38—C33—C34—C35	0.2 (4)
N1—C1—C20—C21	−176.3 (2)	C39—C15—C16—N6	172.2 (2)
N1—C4—C5—C6	5.0 (4)	C39—C15—C16—C17	−11.6 (4)
N1—C4—C5—C27	−176.9 (2)	C39—C40—C41—C42	−1.0 (6)
N2—C6—C7—C8	1.1 (3)	C40—C39—C44—C43	0.0 (6)
N2—C9—C10—C11	4.2 (5)	C40—C41—C42—Cl4	−177.3 (3)
N2—C9—C10—C33	−177.2 (3)	C40—C41—C42—C43	2.3 (6)
N3—C11—C12—C13	−1.1 (3)	C41—C42—C43—C44	−2.4 (7)
N3—C14—C15—C16	1.7 (4)	C42—C43—C44—C39	1.3 (7)
N3—C14—C15—C39	−179.6 (2)	C44—C39—C40—C41	−0.2 (5)
N6—C16—C17—C18	0.5 (3)	C45—C46—C47—C48	1.2 (5)
N6—C19—C20—C1	0.9 (4)	C45—C46—C47—C51	−180.0 (3)
N6—C19—C20—C21	−179.2 (2)	C46—C45—C50—S1	175.8 (2)
C1—N1—C4—C3	1.9 (3)	C46—C45—C50—C49	−1.8 (4)
C1—N1—C4—C5	−176.2 (2)	C46—C47—C48—C49	−0.9 (5)
C1—C2—C3—C4	1.4 (3)	C47—C48—C49—C50	−0.8 (5)
C1—C20—C21—C22	−121.1 (3)	C48—C49—C50—S1	−175.5 (2)
C1—C20—C21—C26	58.4 (3)	C48—C49—C50—C45	2.1 (4)
C2—C1—C20—C19	−174.2 (3)	C50—C45—C46—C47	0.1 (5)
C2—C1—C20—C21	5.9 (4)	C51—C47—C48—C49	−179.7 (3)
C2—C3—C4—N1	−2.1 (3)	K1—O1—C53—C52	−55.6 (2)
C2—C3—C4—C5	176.0 (3)	K1—O1—C54—C55	49.9 (2)
C3—C4—C5—C6	−172.8 (3)	K1—O2—C55—C54	44.9 (3)
C3—C4—C5—C27	5.3 (4)	K1—O2—C56—C57	−59.5 (2)
C4—N1—C1—C2	−1.0 (3)	K1—O3—C59—C58	−46.5 (3)
C4—N1—C1—C20	−179.1 (2)	K1—O3—C60—C61	49.5 (3)
C4—C5—C6—N2	−13.1 (4)	K1—O4—C61—C60	48.1 (3)
C4—C5—C6—C7	161.6 (3)	K1—O4—C62—C63	−62.8 (3)
C4—C5—C27—C28	121.9 (3)	K1—O5—C65—C64	−60.9 (3)
C4—C5—C27—C32	−61.8 (4)	K1—O5—C66—C67	43.5 (3)
C5—C6—C7—C8	−174.3 (3)	K1—O6—C67—C66	50.1 (3)
C5—C27—C28—C29	177.4 (3)	K1—O6—C68—C69	−49.2 (3)
C5—C27—C32—C31	−176.8 (3)	K1—N4—C52—C53	−41.1 (3)
C6—N2—C9—C8	−1.1 (3)	K1—N4—C58—C59	−47.4 (3)
C6—N2—C9—C10	179.7 (3)	K1—N4—C64—C65	−37.0 (3)
C6—C5—C27—C28	−59.8 (3)	K1—N5—C57—C56	−39.3 (3)
C6—C5—C27—C32	116.5 (3)	K1—N5—C63—C62	−36.5 (3)
C6—C7—C8—C9	−1.7 (3)	K1—N5—C69—C68	−44.2 (3)
C7—C8—C9—N2	1.8 (3)	O1—C54—C55—O2	−62.6 (3)
C7—C8—C9—C10	−178.9 (3)	O2—C56—C57—N5	69.7 (3)
C8—C9—C10—C11	−174.9 (3)	O3—C60—C61—O4	−64.1 (3)
C8—C9—C10—C33	3.6 (4)	O4—C62—C63—N5	68.8 (4)
C9—N2—C6—C5	175.3 (3)	O5—C66—C67—O6	−60.8 (3)
C9—N2—C6—C7	0.0 (3)	O6—C68—C69—N5	64.5 (3)
C9—C10—C11—N3	5.9 (5)	N4—C52—C53—O1	67.3 (3)
C9—C10—C11—C12	−172.0 (3)	N4—C58—C59—O3	64.2 (3)
C9—C10—C33—C34	−115.8 (3)	N4—C64—C65—O5	69.0 (3)
C9—C10—C33—C38	58.5 (4)	C52—N4—C58—C59	73.5 (3)
C10—C11—C12—C13	177.1 (3)	C52—N4—C64—C65	−157.8 (2)
C10—C33—C34—C35	174.6 (3)	C53—O1—C54—C55	179.5 (2)
C10—C33—C38—C37	−173.0 (3)	C54—O1—C53—C52	174.4 (2)
C11—N3—C14—C13	1.7 (3)	C55—O2—C56—C57	165.3 (2)
C11—N3—C14—C15	−176.0 (3)	C56—O2—C55—C54	176.8 (2)
C11—C10—C33—C34	62.9 (4)	C57—N5—C63—C62	−154.6 (3)
C11—C10—C33—C38	−122.9 (3)	C57—N5—C69—C68	74.4 (3)
C11—C12—C13—C14	2.0 (3)	C58—N4—C52—C53	−160.5 (2)
C12—C13—C14—N3	−2.4 (3)	C58—N4—C64—C65	81.1 (3)
C12—C13—C14—C15	175.3 (3)	C59—O3—C60—C61	−166.8 (2)
C13—C14—C15—C16	−175.6 (3)	C60—O3—C59—C58	170.7 (2)
C13—C14—C15—C39	3.0 (4)	C61—O4—C62—C63	168.3 (3)
C14—N3—C11—C10	−178.6 (3)	C62—O4—C61—C60	175.3 (3)
C14—N3—C11—C12	−0.4 (3)	C63—N5—C57—C56	80.5 (3)
C14—C15—C16—N6	−9.2 (4)	C63—N5—C69—C68	−165.5 (3)
C14—C15—C16—C17	167.0 (3)	C64—N4—C52—C53	79.0 (3)
C14—C15—C39—C40	−71.5 (4)	C64—N4—C58—C59	−166.1 (2)
C14—C15—C39—C44	108.0 (3)	C65—O5—C66—C67	173.5 (2)
C15—C16—C17—C18	−176.2 (3)	C66—O5—C65—C64	165.0 (2)
C15—C39—C40—C41	179.3 (3)	C67—O6—C68—C69	174.4 (2)
C15—C39—C44—C43	−179.5 (4)	C68—O6—C67—C66	−173.4 (2)
C16—N6—C19—C18	−1.6 (3)	C69—N5—C57—C56	−159.3 (2)
C16—N6—C19—C20	178.9 (2)	C69—N5—C63—C62	84.4 (3)
C16—C15—C39—C40	107.3 (3)	O7—C72—C73—C70	38.8 (10)
C16—C15—C39—C44	−73.3 (4)	C71—O7—C72—C73	−35.2 (10)
C16—C17—C18—C19	−1.5 (3)	C71—C70—C73—C72	−26.3 (9)
C17—C18—C19—N6	2.0 (3)	C72—O7—C71—C70	18.1 (9)
C17—C18—C19—C20	−178.5 (3)	C73—C70—C71—O7	5.1 (8)
C18—C19—C20—C1	−178.5 (3)	O7A—C70A—C71A—C72A	32.9 (15)
C18—C19—C20—C21	1.4 (4)	C70A—O7A—C73A—C72A	−1.3 (14)
C19—N6—C16—C15	177.4 (2)	C70A—C71A—C72A—C73A	−31.9 (16)
C19—N6—C16—C17	0.7 (3)	C71A—C72A—C73A—O7A	16.5 (15)
C19—C20—C21—C22	59.0 (3)	C73A—O7A—C70A—C71A	−22.3 (16)
C19—C20—C21—C26	−121.5 (3)	O8—C74—C75—C76	−28.5 (4)
C20—C1—C2—C3	177.8 (2)	C74—O8—C77—C76	4.2 (5)
C20—C21—C22—C23	179.1 (2)	C74—C75—C76—C77	30.4 (5)
C20—C21—C26—C25	−178.8 (3)	C75—C76—C77—O8	−22.4 (5)
C21—C22—C23—C24	−0.1 (4)	C77—O8—C74—C75	15.8 (4)

## References

[bb1] Adler, A. D., Longo, F. R., Finarelli, J. D., Goldmacher, J., Assour, J. & Korsakoff, L. (1967). *J. Org. Chem.* **32**, 476–476.

[bb2] Adler, A. D., Longo, F. R., Kampas, F. & Kim, J. (1970). *J. Inorg. Nucl. Chem.* **32**, 2443–2445.

[bb3] Bruker (2013). *APEX2*, *SAINT* and *SADABS*. Bruker AXS Inc., Madison, Wisconsin, USA.

[bb4] Byrn, M. P. & Strouse, C. E. (1991). *J. Am. Chem. Soc.* **113**, 2501–2508.

[bb5] Dolomanov, O. V., Bourhis, L. J., Gildea, R. J., Howard, J. A. K. & Puschmann, H. (2009). *J. Appl. Cryst.* **42**, 339–341.

[bb6] Fleischer, E. B. & Srivastava, T. S. (1969). *J. Am. Chem. Soc.* **91**, 2403–2405.

[bb7] Gibson, Q. H., Hoffman, B. M., Crepeau, R. H., Edelstein, S. J. & Bull, C. (1974). *Biochem. Biophys. Res. Commun.* **59**, 146–151. 10.1016/s0006-291x(74)80186-5 4842296

[bb8] He, M., Li, X., Liu, Y. & Li, J. (2016). *Inorg. Chem.* **55**, 5871–5879. 10.1021/acs.inorgchem.6b00173 27228473

[bb9] Hoffman, B. M., Gibson, Q. H., Bull, C., Crepeau, R. H., Edelstein, S. J., Fisher, R. G. & McDonald, M. J. (1975). *Ann. NY Acad. Sci.* **244**, 174–186. 10.1111/j.1749-6632.1975.tb41530.x 1056162

[bb10] Hu, C., Sulok, C. D., Paulat, F., Lehnert, N., Twigg, A. I., Hendrich, M. P., Schulz, C. E. & Scheidt, W. R. (2010). *J. Am. Chem. Soc.* **132**, 3737–3750. 10.1021/ja907584x PMC2846462 20192189

[bb11] Kirner, J. F., Reed, C. A. & Scheidt, W. R. (1977). *J. Am. Chem. Soc.* **99**, 2557–2563. 10.1021/ja00450a025 850028

[bb12] Miller, K. M. & Strouse, C. E. (1984*a*). *Acta Cryst.* C**40**, 1324–1327.

[bb13] Miller, K. M. & Strouse, C. E. (1984*b*). *Inorg. Chem.* **23**, 2395–2400.

[bb14] Shannon, R. D. (1976). *Acta Cryst.* A**32**, 751–767.

[bb15] Sheldrick, G. M. (2015*a*). *Acta Cryst.* A**71**, 3–8.

[bb16] Sheldrick, G. M. (2015*b*). *Acta Cryst.* C**71**, 3–8.

[bb17] Stolzenberg, A. M., Strauss, S. H. & Holm, R. H. (1981). *J. Am. Chem. Soc.* **103**, 4763–4778.

[bb18] Zhao, J., Qian, F., Guo, W., Li, J. & Lin, Z. (2021). *Inorg. Chem.* **60**, 7465–7474. 10.1021/acs.inorgchem.1c00755 33947188

